# Electroencephalogram microstates and functional connectivity of cybersickness

**DOI:** 10.3389/fnhum.2022.857768

**Published:** 2022-08-22

**Authors:** Sungu Nam, Kyoung-Mi Jang, Moonyoung Kwon, Hyun Kyoon Lim, Jaeseung Jeong

**Affiliations:** ^1^Korea Advanced Institute of Science and Technology, Daejeon, South Korea; ^2^Korea Research Institute of Standards and Science, Daejeon, South Korea

**Keywords:** cybersickness, EEG microstate, topographical analysis, electroencephalography, functional connectivity

## Abstract

Virtual reality (VR) is a rapidly developing technology that simulates the real world. However, for some cybersickness-susceptible people, VR still has an unanswered problem—cybersickness—which becomes the main obstacle for users and content makers. Sensory conflict theory is a widely accepted theory for cybersickness. It proposes that conflict between afferent signals and internal models can cause cybersickness. This study analyzes the brain states that determine cybersickness occurrence and related uncomfortable feelings. Furthermore, we use the electroencephalogram (EEG) microstates and functional connectivity approach based on the sensory conflict theory. The microstate approach is a time–space analysis method that allows signals to be divided into several temporarily stable states, simultaneously allowing for the exploration of short- and long-range signals. These temporal dynamics can show the disturbances in mental processes associated with neurological and psychiatric conditions of cybersickness. Furthermore, the functional connectivity approach gives us in-depth insight and relationships between the sources related to cybersickness. We recruited 40 males (24.1 ± 2.3 years), and they watched a VR video on a curved computer monitor for 10 min to experience cybersickness. We recorded the 5-min resting state EEG (baseline condition) and 10-min EEG while watching the VR video (task condition). Then, we performed a microstate analysis, focusing on two temporal parameters: mean duration and global explained variance (GEV). Finally, we obtained the functional connectivity data using eLoreta and lagged phase synchronization (LPS). We discovered five sets of microstates (A–E), including four widely reported canonical microstates (A–D), during baseline and task conditions. The average duration increased in microstates A and B, which is related to the visual and auditory networks. The GEV and duration decreased in microstate C, whereas those in microstate D increased. Microstate C is related to the default mode network (DMN) and D to the attention network. The temporal dynamics of the microstate parameters are from cybersickness disturbing the sensory, DMN, and attention networks. In the functional connectivity part, the LPS between the left and right parietal operculum (OP) significantly decreased (*p* < 0.05) compared with the baseline condition. Furthermore, the connectivity between the right OP and V5 significantly decreased (*p* < 0.05). These results also support the disturbance of the sensory network because a conflict between the visual (V5) and vestibular system (OP) causes cybersickness. Changes in the microstates and functional connectivity support the sensory conflict theory. These results may provide additional information in understanding brain dynamics during cybersickness.

## Introduction

Virtual reality (VR) is evolving rapidly to simulate the real world. Because it is a virtual world, it can simulate various things, including unrealistic things. Thus, researchers have rapidly developed and increased its use for multiple purposes, such as movies, video games, sports, and medical tools for psychiatrists and therapists to treat mental illness (Kim, 2005; Porras et al., 2018).

However, VR still has unresolved problems, such as cybersickness, which is the main obstacle for users and content makers. Watching 3D movies or playing 3D games can trigger cybersickness. Users might experience cybersickness while immersed in VR content when they perceive self-movement caused by optical flow while they are immobile. Its symptoms include sweating, yawning, dizziness, spatial disorientation, instability, fatigue, nausea, and vomiting (McCauley and Sharkey, 1992; Mazloumi Gavgani et al., 2018). Several researchers explained cybersickness using sensory conflict, eye movement hypothesis, subjective vertical conflict, postural instability, and other theories (Laviola, 2000; Gallagher and Ferrè, 2018; Lim et al., 2018).

Among these studies, the most widely accepted cybersickness theory is the sensory conflict theory. It proposes that conflict between afferent signals from visual, vestibular, and somatic sensors and signals from internal models of the central nervous system can cause cybersickness (Zhang et al., 2016). In support of the sensory conflict theory, researchers have demonstrated that a strong illusion of apparent self-movement called vection, generated using visual movement in a wide field of view, often precedes cybersickness (Palmisano et al., 2017). Vection can occur when motion signals from visual inputs collide with stationary signals provided by vestibular and/or proprioceptive inputs. An fMRI study showed that during vection, activities of the human motion-sensitive middle temporal (MT+) area desynchronized between the left and right hemispheres (Miyazaki et al., 2015). Furthermore, this MT area interacts with the parieto-insular vestibular cortex, which is the center of vestibular sensation. Another study showed decreased functional connectivity between MT+/V5 and several other striatal and extra-striatal visual processing regions compared to resting states (Hampson et al., 2004). Together, these results indicate a significant relationship between the visual and vestibular systems, supporting the sensory conflict theory—the theory can explain cybersickness neurophysiologically.

Electroencephalogram (EEG) is commonly used to evaluate cybersickness during VR experiences (Kim et al., 2005; Lim et al., 2021). During an EEG recording, specific topography (or microstate) will persist for 50–100 ms before changing to a different topography (Michel and Koenig, 2018). Thus, we can divide the signals into several temporarily stable states to explore short and long ranges simultaneously. Furthermore, these stable states are highly reproducible within and across participants; a clustering algorithm can group microstates into a finite set of classes based on topographical similarity (Khanna et al., 2014). These microstates offer various parameters with physiological relevance to explain variation across behavioral and psychological states and neuropsychiatric disorders (Al Zoubi et al., 2019; Bréchet et al., 2020; Kalburgi et al., 2020). Also, in contrast to conventional EEG power spectrum analysis techniques that seek general understanding of cybersickness, the microstate analysis leverages global large-scale brain networks to examine complex mental activity. Furthermore, compared to the slow dynamics in fMRI signals, microstate analysis of the EEG offers a better temporal resolution that enables fast changes in global network dynamics of the brain in response to cybersickness on a sub-second time scale, which is a significant advantage over other methods (Michel and Koenig, 2018). Therefore, microstates are an appropriate tool for evaluating the disturbances in mental processes associated with neurological and psychiatric conditions of cybersickness.

Recently, researchers have gained physiological and pathological inspiration by exploring the functional connectivity of the brain using high-density (HD) EEG, which has the advantages of being non-invasive, cheap, and portable (Piano et al., 2017). Because microstate analysis is limited to data on the scalp, finding sources for in-depth analysis and observing the relationships between them could lead to more diverse interpretations. In addition, it was more important in our study because it can overcome certain limitations, such as volume conduction problems; we used HD EEG (257 channel) (Barzegaran and Knyazeva, 2017). A recent study (Li et al., 2021) showed that functional connectivity between the frontal and left parietal regions decreased during cybersickness. Furthermore, the frontal–occipital functional connectivity increased when fully immersed in trained VR (Kang et al., 2021). However, the authors stated limitations, such as small sample size, few EEG channels (5-channel), and a single analysis for cybersickness.

Several studies have reported gender-based differences in motion sickness symptoms, stating that women are generally more sensitive to cybersickness than are men (Paillard et al., 2013; Freitag et al., 2016; Rangelova et al., 2020). An early finding postulated that these differences occur because women have a larger field of view than men (Kennedy and Frank, 1985). Additionally, male subjects were less affected by hormones although they had more VR experiences (Clemes and Howarth, 2005). Although some studies argue that gender differences can be reduced by adjusting the inter-pupillary distance (Stanney et al., 2020), others state otherwise (Hsiao et al., 2019; Melo et al., 2019; Curry et al., 2020). Based on these reasons, gender differences can affect the result and we must consider that factor carefully.

Our research group planned a long-term study to investigate the severity of cybersickness in stages caused by human factors, such as gender and age. Thus, we would like to compare the following EEG scenarios: in a resting state, during exposure to simple content using a curved monitor, and during exposure to simple and complex contents while wearing a head-mounted display (HMD). This comparison is to investigate and quantify the brain dynamics caused by the severity of cybersickness. Then, we planned to investigate the characteristics of cybersickness from a wider variety of people of different genders and ages and compare them with those previously studied. First, using simple content consisting of land and sky displayed on a curved monitor, we investigated changes in male participants affected by cybersickness (in their 20s) in EEG.

This study used microstates analysis and source connectivity to understand the large-scale brain networks during cybersickness. These dynamic characteristics are crucial for understanding complex brain cognition and behavior (Liao et al., 2017). Furthermore, they can cause the development of new diagnostic or monitoring tools (Dennison et al., 2016). However, previous studies did not show the microstates of cybersickness, which can provide further insight into exploring cybersickness.

## Materials and methods

### Participants

We recruited 40 male participants (24.1 ± 2.3 years) for the cybersickness EEG test. Based on a previous study conducted by our research group, we can divide the participants using the MSSQ, which is a self-report questionnaire measuring susceptibility to motion sickness (Lim et al., 2021). The mean score was 21.56, with 20 participants scoring higher than the mean score and others scoring lower. The subjects were right-handed and healthy, and they did not take any medication. All subjects participated in this study after signing a prior agreement. The Institutional Review Board (IRB) of the Korea Advanced Institute of Science and Technology and the Ethical Committee of the Korea Research Institute of Standards and Science (KRISS) approved the experimental procedures in this study (KH2020-145, KRISS-IRB-2021-03). The participants provided informed written consent.

### Evaluation index

**FIGURE 1:**
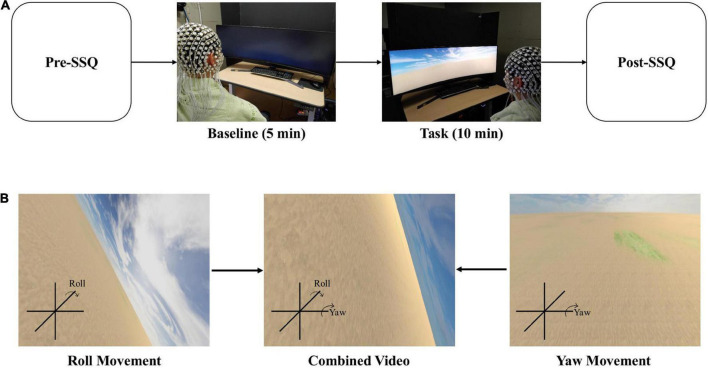
EEG recordings during cybersickness condition. **(A)** Experimental scenario. **(B)** Scenes could be changed to display the roll and yaw motion. The participant will watch the combined video through a curved monitor.

The simulation sickness questionnaire (SSQ) is a survey for describing simulator sickness by evaluating 16 symptoms on a 4-point scale (0–3). Based on factor analysis, these symptoms were divided into three general categories: oculomotor, disorientation, and nausea (Kennedy et al., 1993). SSQ measures visually induced cybersickness by assessing symptoms of cybersickness. The participants answered the SSQ twice: before watching VR video content (Supplementary Video 1) (named Pre-SSQ) for a baseline condition and after watching VR video content (named Post-SSQ) for the task condition (Figure 1A). The figure compares the two SSQ results.

### Experimental protocol

#### Test procedure

We divided the experimental protocol into two conditions: baseline and task. The baseline condition checks the resting state of EEG, while the task condition is designed to cause cybersickness. With the eyes opened, the baseline condition lasted for 5 min without action. Then, the participants watched a VR video for 10 min to induce cybersickness during the task condition (Figure 1A). Finally, the participants completed the SSQ to measure cybersickness before the baseline and after the task conditions.

#### Apparatus and stimuli

We created a 10-min VR video as 10 min is enough to induce cybersickness (Sharples et al., 2008). The distance between the participants and the curved monitor that presented the VR video was 65 cm. Additionally, the height of all participants’ chairs was adjusted to fit their eyes in the center of the monitor. A screen fence blocked the view behind the curved monitor. The field of view was controlled for all observers to avoid peripheral surroundings that act as stable landmarks (Kim and Kim, 2019). Through curved computer monitors (Samsung, 120 × 34 cm), the scene viewed by the participants constantly moved back and forth at a speed of 20 m/s, and the rotational angular velocity pseudo-randomly changed every minute rather than sequentially (15°/s–20 m/s, 30°/s–20 m/s, 45°/s–20 m/s, 60°/s–20 m/s, 75°/s–20 m/s, 90°/s–20 m/s, 105°/s–20 m/s, 120°/s–20 m/s, 135°/s–20 m/s, and 150°/s–20 m/s). For example, a participant’s field of view moved forward at a speed of 20 m/s and changed the yaw and roll (Figure 1B) at a speed of 30°/s for the first 30 s, and it moved backward at the same movement and angular speed for the next 30 s. This 1-min video was repeated pseudo-randomly 10 times (for 10 min) with a change in angular velocity. We used the Unity program to create the VR scenes in this study. Several parameters, such as rotational speed, linear movement speed, motion axis, background complexity, and scene fidelity, can affect cybersickness. Thus, we tried to keep the condition as simple as possible to minimize the impact of other variables.

#### Electroencephalogram recordings

The EEG signals were collected in the electric shielding chamber using a 256-channel HydroCel Geodesic Sensor Net (Electrical Geodesics Incorporated, Eugene, OR, United States). We referenced all electrodes to Cz, and impedance remained below 50 kΩ (Tucker et al., 2003). We recorded EEG using a 0.1–100-Hz band pass at a sampling rate of 1000 Hz. We analyzed EEG data using EEGLAB, which is an open-source toolbox operated in MATLAB (MathWorks, Natick, MA, United States). Furthermore, we used an ADJUST (an automatic EEG artifacts detector based on the joint use of spatial and temporal features) plug-in with EEGLAB to remove EEG artifacts. We applied a 1-Hz high-pass filter in the EEG data pre-processing and a 40-Hz low-pass filter. After filtering, we downsampled signals to 125 Hz and re-referenced them using a common average reference. To eliminate ocular (eye blinks, vertical eye movements, and horizontal eye movements) and muscular artifacts, we first applied independent component analysis (ICA) to the data (as implemented in the EEGLAB toolbox) (Vialatte et al., 2008; McMenamin et al., 2010). Then, we used the ADJUST plug-in to remove the ICA components that contained the artifacts based on the default plug-in criteria (Mognon et al., 2011). We selected epochs (4 min) in the middle of both conditions for this study.

#### Electroencephalogram microstates analysis

The following steps must be taken after data pre-processing: (1) find the most representative maps from EEG data and (2) fit these maps back into the EEG data and calculate temporal parameters and statistics from it.

In this process, the segmentation stage is performed using two spatial cluster analyses based on a modified version of the k-means algorithm to find the optimal topography set for microstate analysis (Pascual-Marqui et al., 1995). First, it was performed at the individual level for each participant, and then it was performed at the global level for all participants. Global field power was calculated from each epoch to find the most dominant map for each participant. We ignored the polarity of the map while using this clustering method. To obtain the optimal number of clusters, we used a meta-criterion that combines seven independent optimization criteria (Pascual-Marqui et al., 1995; Charrad et al., 2014). Further, to perform at the global level, the dominant maps for all participants (baseline, task) were submitted together to a clustering algorithm to find the most representative maps across subjects. The number of global maps was selected based on the same meta-criterion, which was used at the individual level, thus resulting in the five microstate maps in this study (polarity of the map was ignored as in the individual level). The microstate maps were fitted back into the individual EEG data to define the microstates after they were selected. Each time frame of EEG data was labeled with one of five microstate maps, which was identified during global level clustering analysis, by calculating the spatial correlations (winner-takes-all, and only the correlation above 0.5 was selected) (Pascual-Marqui et al., 1995). Furthermore, we calculated two temporal parameters for each participant: GEV and duration. The duration indicates how long it lasts between each microstate class in milliseconds. The GEV indicates how well the microstate topographies explain the original EEG data. We used the free Cartool (Brunet et al., 2011) software 3.91, programmed by Denis Brunet Cartool, to perform the microstate analysis. The software also includes a meta-criterion calculation.

#### Electroencephalogram source connectivity

First, we used eLORETA with the LORETA KEY software for source localization (Pascual-Marqui, 2007a,b; Pascual-Marqui et al., 2011). We performed the eLORETA computations in a realistic head model using the MNI152 (Montreal Neurological Institute 152) template (Mazziotta et al., 2001; Fuchs et al., 2002) with the 3D solution space restricted to 6,000 voxels of 5-mm^3^ resolution, which comprises cortical gray matter determined using the probabilistic Talairach atlas (Lancaster et al., 2000). Next, we applied coordinates of electrode positions in the MNI152 scalp to a digitized MRI version of the Talairach atlas (McConnel Brain Imaging Centre, Montreal Neurological Institute, McGill University). We used the resulting coordinates to compute the eLORETA transformation matrix. Furthermore, we defined the region of interest (ROI). We extracted 10 ROIs (Table 1) from the visual and vestibular system parts, which interact when experiencing motion sickness (zu Eulenburg et al., 2012; Miyazaki et al., 2021). We used the single nearest voxel option as the spatial resolution of the eLORETA was low, and the single centroid voxel was the best representative of the ROI. For the functional connectivity analysis for each pair of ROIs, we used a lagged phase synchronization (LPS) to ignore the instantaneous component of total connectivity and considered only the lagged component (Pascual-Marqui, 2007a,b; Jahng et al., 2017).

**TABLE 1:** Region of interest (ROI).

ROI	MNI	Structure
1	10 −42 6	Right V1
2	−16 −68 8	Left V1
3	36 −72 18	Right MT+/V5
4	−42 −70 10	Left MT+/V5
5	−36 0 −4	Central insula
6	−52 2 2	OP4
7	−46 −14 12	OP1
8	−38 −20 16	OP2
9	48 4 50	Premotor cortex
10	−40 −16 14	Posterior insula

### Statistical analysis

We analyzed SSQ with descriptive statistics using SPSS 21 software. We performed a paired *t*-test to investigate the condition differences in temporal parameters from each microstate. Furthermore, we performed a paired *t*-test to determine the difference in LPS between the two conditions. The variance smoothing parameter and randomization number were 0 and 5,000, respectively. We created a file containing the maximal thresholds, and the thresholds at probability values of *p* < 0.01, *p* < 0.05, and *p* < 0.01 (Friston et al., 1991). Finally, we used those values to show the significant changes in functional connectivity.

## Results

**FIGURE 2:**
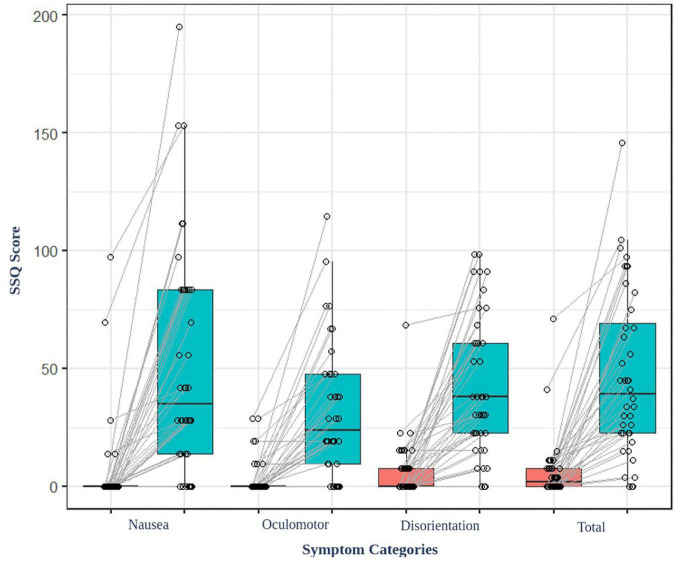
SSQ score of the participant before and after watching the VR video content. All the participants felt cybersickness (*p* < 0.001).

The result of the average SSQ score before (baseline) and after (task) watching VR content showed significant differences (*t* = −6.85, *p* < 0.001) (Figure 2). Additionally, the participants felt cybersickness. There were no significant associations between the parameters of the EEG and the degree of cybersickness based on SSQ sub-categories (total, nausea, oculomotor, and disorientation etc.). There were no significant associations between the functional connectivity and the GEV or duration of microstates either.

**FIGURE 3:**
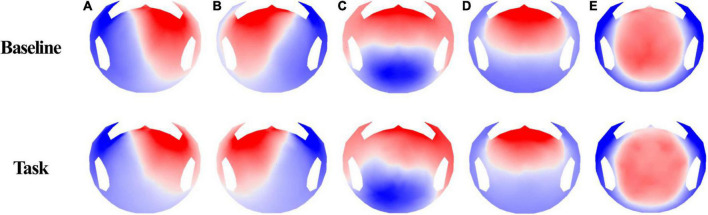
EEG microstate topographies for both conditions (base, task).

Microstate segmentation of 40 participants recorded using 256-channel EEG showed five microstate topographies (Figure 3), which were consistent across conditions. These five microstate classes across participants explained 78.76 and 80.93% of the global variance in the resting and task conditions, respectively. Additionally, five microstate maps showed a high spatial correlation of the topographies ranging from 0.94 to 0.98. The four microstates (A–D) obtained in the two conditions were similar to the four class models maps consistently identified in the literature. Microstates A and B have topographic map fields with diagonal axis orientations, C has anterior–posterior orientations, and D has fronto-central extreme locations (Michel and Koenig, 2018). Microstate E also had a topography similar to that of several studies (Custo et al., 2017; Bréchet et al., 2020).

**FIGURE 4:**
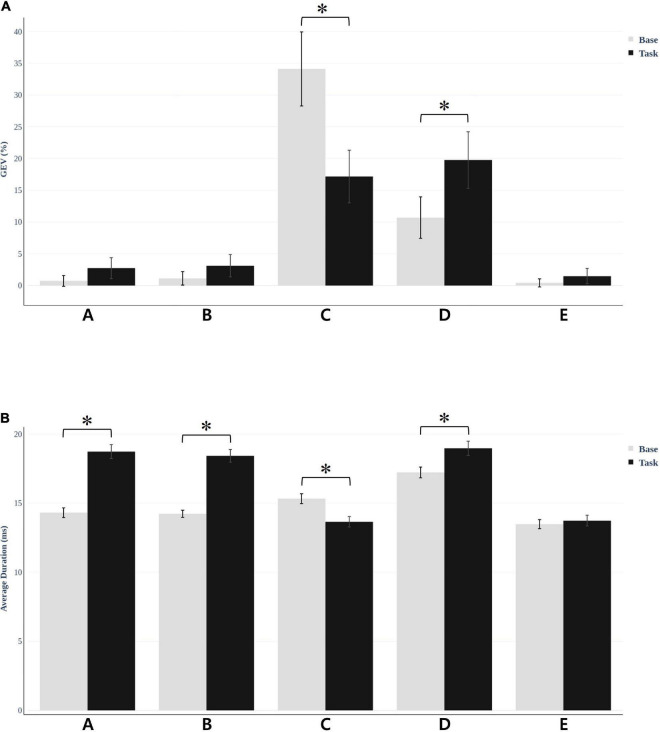
Microstate global explained variance (GEV) and average duration was different for each condition. **(A)** For resting vs. task condition, microstate C decreased (*t* = −6.156, *p < 0.001) and D increased (*t* = −8.53, *p < 0.001) in GEV, which was statistically significant. **(B)** The average duration of the microstates under the two conditions. The results were significant in all microstates except E.

We fitted back five main maps from the baseline and task conditions to each participant’s original EEG using special correlation analysis and winner-takes-all labeling. This fitting method allowed us to uninterruptedly calculate the duration of each microstate and the global variance explained by each map (GEV). Researchers commonly use these parameters to show the spatiotemporal properties of EEG microstates. Microstate duration and GEV were different for each condition. For the resting vs. task condition, microstate C decreased (*t* = −6.156, *p* < 0.001) and D increased (*t* = −8.53, *p* < 0.001), which was statistically significant (Figure 4A). There were no statistically significant values for the other microstates. Furthermore, we compared the average duration of the microstates under the two conditions (Figure 4B). The other microstate showed significant change except for microstate E. Microstates A, B, and D (*t* = −7.086, *p* < 0.001; *t* = −8.118, *p* < 0.001; and *t* = 6.482, *p* < 0.001, respectively) showed increased values, while microstate C showed decreased values (*t* = −5.230, *p* < 0.001). Microstate E showed no significant difference for either GEV (*t* = −1.067, *p* = 0.293) and duration (*t* = −1.051, *p* = 0.300).

We observed statistical significance in the LPS when comparing the baseline condition with the task condition; the threshold for significance was *t* = 2.187 (corresponding to *p* < 0.05). The functional connectivity between the left parietal operculum (OP1) and right parietal operculum (OP2) decreased. In addition, connectivity between OP2 and the right medial temporal area (V5) decreased. There were no significant associations between the functional connectivity and the GEV or duration of microstates (all absolute *r*-values < 0.45).

## Discussion

This study analyzes the neural mechanisms of cybersickness. To achieve this, we checked microstates and functional connectivity under cybersickness. Our main findings are as follows: (1) there are significantly increased and decreased values of microstate parameters under cybersickness and (2) cybersickness decreases functional connectivity between the visual and vestibular systems.

We selected five maps for microstate analysis. A, B, C, and D are common microstates among these maps, and several studies also referred to E (Custo et al., 2017; Michel and Koenig, 2018; Bréchet et al., 2020). Thus, we investigated microstates A, B, C, and D, which showed significant changes in GEV and duration.

Before discussing the parameter change, it is crucial to understand the sources of each microstate. The source of a microstate is vital in interpreting parameter changes and explaining cybersickness. An fMRI study discovered sources of resting states in microstates (Custo et al., 2017). At first, microstate A showed strong activity in the left, middle, and superior temporal lobes, including Brodmann areas 41 (BA41, primary auditory cortex) and 22 (BA22, Wernicke), and the left insular cortex. Microstate B showed activity in the left and right occipital cortices (cuneus), including Brodmann areas 17 and 18 (primary visual cortex). The posterior cingulate, right insula, and the temporal cortex were also sources of microstate B. Then, microstate C showed strong activation in the precuneus and the posterior cingulate cortex. A second weaker activated area was the left angular gyrus. Lastly, microstate D showed strong activation in the right inferior parietal lobe (BA40) and the right, middle, and superior frontal gyri. The right insula (BA13) was also activated (Bréchet et al., 2019). These aforementioned and other fMRI studies show that microstate A is related to the auditory network, microstate B to the visual network, microstate C to the saliency network, and microstate D to the attention network (Britz et al., 2010).

Microstates A and B, which are related to auditory and visual networks, showed an increase in average duration in our study. Similarly, according to the microstate analysis result of relapsing-remitting multiple sclerosis using HD EEG, patients showed an increased average duration of microstates A and B compared with healthy controls (Gschwind et al., 2016). It was possible to predict the duration of the disease and the annual relapse rate using the two changes of the microstates and apply a stepwise multiple linear regression model. This finding suggests that multiple sclerosis affects the sensory network (visual and auditory), which is consistent with the fMRI studies (Britz et al., 2010). We can also interpret that the increased duration in microstates A and B in our study occurred for a similar reason. Therefore, we can assume that disturbance of the sensory network caused by cybersickness increased A and B durations.

We discovered a decreased average duration and GEV during cybersickness. In addition, microstate C decreased in the visualization condition (Milz et al., 2016) and high cognitive processes, particularly math problems (Bréchet et al., 2019). We also found an association between microstate C and the DMN; additional fMRI studies supported these findings (Damoiseaux et al., 2008; Lei et al., 2014). According to these findings, microstate C is a task-negative network with decreasing activity during the performance of cognitive works. These findings are also related to worse cognitive status and agree with the neural mechanism issue of cybersickness (Zeng et al., 2015). Thus, a decrease in GEV and duration could result in stress from viewing VR video and disturbance of the DMN caused by motion sickness, as confirmed in the studies described above.

However, microstate D showed a relatively increased duration and GEV than microstate C in our study. Furthermore, we observed increased duration and occurrence when participants engaged in a subtraction task (Seitzman et al., 2017). This result suggests an association between microstate D and the dorsal attention network. However, according to a behavioral manipulation study, it increased during rest rather than performing a goal-directed task (Milz et al., 2016). This discovery suggests an association between microstate D and the reflexive aspects of attention, focus switching, and reorientation. It is also associated with wakefulness and reality testing, which increases during meditation (Faber et al., 2014). However, it decreases during schizophrenia (Lehmann et al., 2005) and under hypnosis (Katayama et al., 2007). Furthermore, watching a VR video that moves continuously and rotates makes participants repeat reorientation and focus switching despite cybersickness. They become more aroused simultaneously. Thus, D also increased in our study.

**FIGURE 5:**
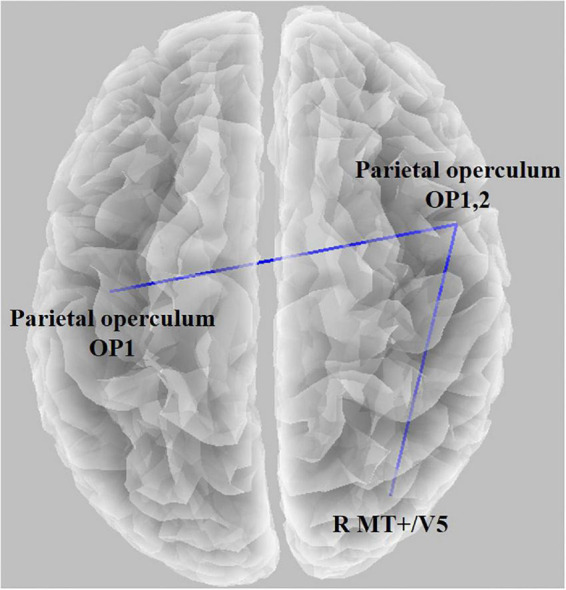
Functional connectivity result using lagged phase synchronization when comparing the baseline condition with the task condition (*t* = 2.187, *p* < 0.05). The functional connectivity between the left parietal operculum (OP1) and right parietal operculum (OP2) decreased, and connectivity between OP2 and the right medial temporal area (V5) decreased.

In the microstate analysis, the sensory network was affected when the duration of microstates A and B increased. Therefore, we wanted to check if it appeared in functional connectivity. Thus, the connectivity between V5 and OP decreased, and the left and right OPs decreased (Figure 5). By viewing moving concentric circles, functional connectivity between MT/V5 and other visual processing brain regions decreased compared with resting states (Hampson et al., 2004). However, several studies discovered that the connectivity between the left and right MT decreases when feeling cybersickness (Miyazaki et al., 2015), and the left and right vestibular also decreases (Li et al., 2021). Additionally, a study confirmed a reduced visual–vestibular interaction in monkeys using visual motion stimulation (Chen et al., 2011), and nausea reduced connectivity between the right and left V1 (Toschi et al., 2017). The tendency to feel motion sickness decreased with a lowered gap between the left and right using a simulator and tDCS (Arshad et al., 2015; Ahn et al., 2020). Therefore, our study showed that cybersickness reduced the connectivity of the visual–vestibular network as confirmed earlier by the microstate analysis, which is consistent with the results of previous studies.

## Conclusion

In this study, we analyzed the microstate and functional connectivity during cybersickness. We discovered changed microstate parameters under the cybersickness conditions and decreased functional connectivity between visual and vestibular networks. These results provide understanding and information about brain dynamics, which change during cybersickness. To the best of our knowledge, this is the first attempt to analyze the brain states of cybersickness in terms of large-scale brain networks on a sub-second time scale (microstates). We clearly showed that the brain states of cybersickness exhibited specific spatiotemporal patterns of the EEG and could be expressed as a superposition of EEG microstates. This indicates that cybersickness potentially affects global brain dynamics in sub-second time scale. We suggest that these distinct temporal dynamics of EEG microstates can be a potential biomarker for detection of cybersickness in various fields including fighter pilots and flight attendants. However, only men participated because of the limitations of this study. We need further research for the localization and discovery of detailed neural mechanisms; however, this study is a promising start in improving our understanding of cybersickness.

## Limitation of the study

This study used simple VR video instead of complex content, such as vertical objects (trees, buildings, etc.). However, using more complex video content would cause differences in the microstate values and network measures. Furthermore, we did not use HMD in this study, which provides more immersive VR content. Future research might identify differences between this study and more immersed stereoscopic content using HMD. One of the significant limitations in the current study is that we only included male participants in the experiment. Thus, the findings obtained from the current study are only relevant for males. We should perform the same protocols of experiments and analyses using both male and female participants in our further investigation to confirm the results. We suggest that the gender-balanced study would clearly show how gender affects the spatiotemporal microstate patterns of brain dynamics in EEGs induced by cybersickness. Furthermore, EEG recordings were made during the baseline and while watching the VR video. Other studies that followed a similar protocol as ours have suggested that decreased alpha power is a sign of cybersickness (Naqvi et al., 2015; Liu et al., 2017). However, several studies have reported that alpha power increases after watching a stimulus (Chuang et al., 2016; Wawrzyk et al., 2018). Thus, EEG results may vary depending on whether it is presenting a visual stimulus or whether it is measured after the visual stimulation disappears. In this study, we measured participants while they were watching VR videos. In the follow-up study, we must check the EEG recording of the resting period after watching the VR video. Additionally, fMRI studies might discover the exact source of cybersickness. Thus, we could have more detailed and drastic results due to more severe cybersickness and enriched information.

## Data availability statement

The raw data supporting the conclusions of this article will be made available by the authors, without undue reservation.

## Ethics statement

The studies involving human participants were reviewed and approved by the Institutional Review Board (IRB) of KAIST and the Ethical Committee of “Korea Research Institute of Standards and Science (KRISS).” The patients/participants provided their written informed consent to participate in this study.

## Author contributions

SN contributed to the design, conceptualization, interpretation of the data, and writing of the original draft. K-MJ and MK provided data acquisition and expertise on EEG pre-processing. HL and JJ provided expertise on EEG microstates and functional connectivity. All authors contributed to the interpretation of the results, manuscript revision, and read and approved the submitted version.
